# Supplementary material for: Humans can use positive and negative spectrotemporal correlations to detect rising and falling pitch
Source: Nat Hum Behav. 2026 Feb 9;10(2):417–33. doi: 10.1038/s41562-025-02371-7 (PMC12932110; doi:10.1038/s41562-025-02371-7)
Supplement: Supplementary file 1 — Reporting Summary [file 41562_2025_2371_MOESM1_ESM.pdf]

nature portfolio

Corresponding author(s): S McDougle & D Clark

Last updated by author(s): Oct 10, 2025

Reporting Summary

Nature Portfolio wishes to improve the reproducibility of the work that we publish. This form provides structure for consistency and transparency in reporting. For further information on Nature Portfolio policies, see our Editorial Policies and the Editorial Policy Checklist.

Statistics

For all statistical analyses, confirm that the following items are present in the figure legend, table legend, main text, or Methods section.

n/a	Confirmed	
☐	☒	The exact sample size (n) for each experimental group/condition, given as a discrete number and unit of measurement
☒	☐	A statement on whether measurements were taken from distinct samples or whether the same sample was measured repeatedly
☐	☒	The statistical test(s) used AND whether they are one- or two-sided<br>*Only common tests should be described solely by name; describe more complex techniques in the Methods section.*
☐	☒	A description of all covariates tested
☒	☐	A description of any assumptions or corrections, such as tests of normality and adjustment for multiple comparisons
☐	☒	A full description of the statistical parameters including central tendency (e.g. means) or other basic estimates (e.g. regression coefficient) AND variation (e.g. standard deviation) or associated estimates of uncertainty (e.g. confidence intervals)
☒	☐	For null hypothesis testing, the test statistic (e.g. F, t, r) with confidence intervals, effect sizes, degrees of freedom and P value noted<br>*Give P values as exact values whenever suitable.*
☒	☐	For Bayesian analysis, information on the choice of priors and Markov chain Monte Carlo settings
☒	☐	For hierarchical and complex designs, identification of the appropriate level for tests and full reporting of outcomes
☒	☐	Estimates of effect sizes (e.g. Cohen's d, Pearson's r), indicating how they were calculated

Our web collection on statistics for biologists contains articles on many of the points above.

Software and code

Policy information about availability of computer code

Data collection	Custom code was written to collect data in PsychToolbox 3.0.18 and run in Matlab 2021b. It is available here: https://github.com/ClarkLabCode/humanAuditoryCorrelations and here: https://doi.org/10.5061/dryad.hmgqnk9w8.
Data analysis	Custom code was written to analyzed psychophysics data. It is available here: https://github.com/ClarkLabCode/humanAuditoryCorrelations and here: https://doi.org/10.5061/dryad.hmgqnk9w8. fMRI code was analyzed using JupyterLab and Nilearn packages. It is available here: https://doi.org/10.5061/dryad.hmgqnk9w8.

For manuscripts utilizing custom algorithms or software that are central to the research but not yet described in published literature, software must be made available to editors and reviewers. We strongly encourage code deposition in a community repository (e.g. GitHub). See the Nature Portfolio guidelines for submitting code & software for further information.

Data

Policy information about availability of data

All manuscripts must include a data availability statement. This statement should provide the following information, where applicable:

- Accession codes, unique identifiers, or web links for publicly available datasets
- A description of any restrictions on data availability
- For clinical datasets or third party data, please ensure that the statement adheres to our policy

All psychophysics data is freely available through the links in the paper. Raw fMRI data is available from the Lead Authors on request.

Research involving human participants, their data, or biological material

Policy information about studies with human participants or human data. See also policy information about sex, gender (identity/presentation), and sexual orientation and race, ethnicity and racism.

Reporting on sex and gender	Participant sex is reported.
Reporting on race, ethnicity, or other socially relevant groupings	na
Population characteristics	Age statistics are reported.
Recruitment	Participants were recruited by the first author from among students at the University.
Ethics oversight	The study was run under a protocol approved by the Yale Institutional Review Board.

Note that full information on the approval of the study protocol must also be provided in the manuscript.

Field-specific reporting

Please select the one below that is the best fit for your research. If you are not sure, read the appropriate sections before making your selection.

☐ Life sciences ☒ Behavioural & social sciences ☐ Ecological, evolutionary & environmental sciences

For a reference copy of the document with all sections, see nature.com/documents/nr-reporting-summary-flat.pdf

Behavioural & social sciences study design

All studies must disclose on these points even when the disclosure is negative.

Study description	Data are quantitative.
Research sample	Yale University students.
Sampling strategy	Sampling was not random; participants were recruited by the authors.
Data collection	A laptop computer recorded psychophysical decisions. A 3T fMRI machine collected fMRI data.
Timing	Spring and Fall 2022, and Spring 2023
Data exclusions	Data were excluded as noted in the methods.
Non-participation	No participants began but did not finish the experiments.
Randomization	There was no assignment to experimental and control groups.

Reporting for specific materials, systems and methods

We require information from authors about some types of materials, experimental systems and methods used in many studies. Here, indicate whether each material, system or method listed is relevant to your study. If you are not sure if a list item applies to your research, read the appropriate section before selecting a response.

Materials & experimental systems

n/a	Involved in the study	
☒	☐	Antibodies
☒	☐	Eukaryotic cell lines
☒	☐	Palaeontology and archaeology
☒	☐	Animals and other organisms
☒	☐	Clinical data
☒	☐	Dual use research of concern
☒	☐	Plants

Methods

n/a	Involved in the study	
☒	☐	ChIP-seq
☒	☐	Flow cytometry
☐	☒	MRI-based neuroimaging

Plants

Seed stocks

na

Novel plant genotypes

na

Authentication

na

Magnetic resonance imaging

Experimental design

Design type

No active tasks were assigned. Data was collected to measure passive responses to specific auditory stimuli, presented as described in the methods.

Design specifications

slow event related design; Fifteen auditory stimuli were presented per run in an event-related design (5 each of three stimulus types: rising, falling, and summed). Each stimulus lasted for 13.33 s, separated by an inter-trial interval (ITI) of 4 s. The order of the three stimulus types was randomized in each run. Participants passively listened to the tones and were not required to render any responses.

Behavioral performance measures

None during fMRI.

Acquisition

Imaging type(s)

Functional and antomical

Field strength

3T

Sequence & imaging parameters

Whole-brain imaging was performed at the Brain Imaging Center at Yale University, on a Siemens 3 T Prisma MRI scanner using a 32-channel head coil. Functional data were acquired with a gradient-echo echoplanar pulse sequence (TR = 0.80 s, TE = 30 ms, flip angle = 52,̊ voxel size = 2.4 mm × 2.4 mm × 2.4 mm, MB acc. factor = 6). T1-weighted MP-RAGE anatomical images were collected as well (TR = 2.5 s, TE = 2.0 ms, flip angle = 8,̊ 208 slices, voxel size = 1.0 mm isotropic).

Area of acquisition

Whole brain scan.

Diffusion MRI ☐ Used ☒ Not used

Preprocessing

Preprocessing software

The fMRI-Prep toolbox was used for preprocessing (Esteban et al. 2019). The anatomical image was corrected for intensity non-uniformity (INU) with N4BiasFieldCorrection (Tustison et al. 2010) and used as T1w-reference. The T1w-reference was then skull-stripped with a Nipype implementation of the antsBrainExtraction.sh workflow in ANTs, and tissue segmentation of cerebrospinal fluid (CSF), white-matter (WM), and gray-matter (GM) was performed on the brain-extracted T1w using FFAST (FSL 6.0.5) (Zhang et al. 2001). Volume-based spatial normalization to standard (MNI) space was performed through nonlinear registration with antsRegistration (ANTs 2.3.3). For each of the BOLD runs, a reference volume and its skull-stripped version were generated using a custom methodology of fMRIPrep. Head-motion parameters were estimated using MCFLIRT (FSL 6.0.5) (Jenkinson et al. 2002) and BOLD time-series were resampled into native space by applying the transforms to correct for head-motion, and the BOLD reference was co-registered to the anatomical reference using mri_coreg (FreeSurfer) followed by FLIRT. Co-registration was configured with 6 DOF. Several confounding time-series were calculated based on the preprocessed BOLD: framewise displacement (FD), DVARS and three region-wise global signals. The BOLD time-series were resampled into standard space, and volumetric resamplings were performed using ANTs.

Normalization

as above: The anatomical image was corrected for intensity non-uniformity (INU) with N4BiasFieldCorrection (Tustison et al. 2010) and used as T1w-reference. The T1w-reference was then skull-stripped with a Nipype implementation of the antsBrainExtraction.sh workflow in ANTs, and tissue segmentation of cerebrospinal fluid (CSF), white-matter (WM), and gray-matter (GM) was performed on the brain-extracted T1w using FFAST (FSL 6.0.5) (Zhang et al. 2001). Volume-based spatial normalization to standard (MNI) space was performed through nonlinear registration with antsRegistration (ANTs 2.3.3).

Normalization template

MNI

Noise and artifact removal

Confound regressors of no interest (generated using fMRIPrep, see above) were entered into each GLM. These included six standard motion regressors, the framewise displacement time course, and white matter and global signal time courses.

Volume censoring | na

Statistical modeling & inference

Model type and settings | Our main analyses involved constructing general linear models (GLMs) to quantify the effects of the three stimulus types within auditory cortex. Univariate GLM analyses were performed using Nilearn (Abraham et al. 2014). Confound regressors of no interest (generated using fMRIPrep, see above) were entered into each GLM. These included six standard motion regressors, the framewise displacement time course, and white matter and global signal time courses. Each stimulus type (rising, falling, and summed) was modeled using boxcar regressors over the entire stimulus presentation phase (13.33 s) of the relevant trials, and was convolved with the canonical double-gamma hemodynamic response function. The main contrast of interest at the group and individual levels compared BOLD responses to the non-summed directional stimuli (i.e., rising and falling) to the summed stimuli (i.e., superimposed rising + falling). The contrast was designed to highlight deviations from a null hypothesis of equivalent responses between directional and opponent stimuli.

Effect(s) tested | Univariate contrast of directional pitch stimuli trials versus combined opponent stimuli

Specify type of analysis: ☐ Whole brain ☒ ROI-based ☐ Both

Anatomical location(s) | We used a probabilistic atlas to restrict analysis to broad temporal auditory areas, as described in the methods.

Statistic type for inference | cluster-wise

(See Eklund et al. 2016)

Correction | non-parametric/permutation tests

Models & analysis

n/a	Involved in the study
☒	☐ Functional and/or effective connectivity
☒	☐ Graph analysis
☐	☒ Multivariate modeling or predictive analysis

Multivariate modeling and predictive analysis | standard univariate GLMs
